# Spatial Distribution and Risk Assessment of Antibiotics in 15 Pharmaceutical Plants in the Pearl River Delta

**DOI:** 10.3390/toxics11040382

**Published:** 2023-04-17

**Authors:** Yuanfei Liu, Xiaoxia Shi, Xiaoxia Chen, Ping Ding, Lijuan Zhang, Jian Yang, Jun Pan, Yunjiang Yu, Jinhua Wu, Guocheng Hu

**Affiliations:** 1 State Environmental Protection Key Laboratory of Environmental Pollution Health Risk Assessment, South China Institute of Environmental Sciences, Ministry of Ecology and Environment, Guangzhou 510535, China; 2 School of Environment and Energy, South China University of Technology, Guangzhou 510641, China; 3 Chongqing Key Laboratory of Water Environment Evolution and Pollution Control in Three Gorges Reservoir, Chongqing Three Gorges University, Chongqing 404000, China

**Keywords:** pharmaceutical plants, antibiotics, removal efficiency, regional distribution, risk assessment

## Abstract

Pharmaceutical plants are an essential source of antibiotics emitted into the aqueous environment. The monitoring of target antibiotics in pharmaceutical plants through various regions is vital to optimize contaminant release. The occurrence, distribution, removal, and ecological risk of 30 kinds of selected antibiotics in 15 pharmaceutical plants in the Pearl River Delta (PRD) were investigated in this study. Lincomycin (LIN) showed the highest concentration (up to 56,258.3 ng/L) in the pharmaceutical plant influents from Zhongshan city. Norfloxacin (NFX) showed a higher detection frequency than other antibiotics. In addition, the spatial distribution of antibiotics in pharmaceutical plants showed significant differences, with higher concentrations of total antibiotics found in pharmaceutical plant influents in Shenzhen City than those of different regions in PRD. The treatment processes adopted by pharmaceutical plants were commonly ineffective in removing antibiotics, with only 26.7% of antibiotics being effectively removed (average removal greater than 70%), while 55.6% of antibiotics had removal rates of below 60%. The anaerobic/anoxic/oxic (AAO)-membrane bioreactor (MBR) combined process exhibited better treatment performance than the single treatment process. Sulfamethoxazole (SMX), ofloxacin (OFL), erythromycin-H_2_O (ETM-H_2_O), sulfadiazine (SDZ), sulfamethazine (SMZ), norfloxacin (NFX), and ciprofloxacin (CIP) in pharmaceutical plant effluents posed high or moderate ecological risk and deserve particular attention.

## 1. Introduction

Antibiotics are pharmacologically and biosynthetically active chemicals that inhibit the growth of microorganisms by acting on the metabolic processes of bacteria, fungi, and protozoa, thereby protecting humans and animals from disease [1,2,3]. The high consumption and non-biodegradable properties of antibiotics lead to a wide distribution of target compounds in different environments [4,5,6,7,8].

Importantly, as antibiotics increasingly enter the nearby environment, prolonged exposure to antibiotics can promote the development of antibiotic resistant bacteria (ARBs) and antibiotic-resistant genes (ARGs), which reduces their therapeutic potential against bacterial pathogens [2,9]. ARG is considered one of the major threats to human and animal health in the 21st century [10]. Therefore, antibiotics have received much attention as an emerging pollutant in the water environment in recent years [11]. China is the largest producer and consumer of antibiotics in the world [12]. The total consumption of antibiotics in China were reported to be 162 kt, much higher than those in other developed countries [12]. As consumption increases, various antibiotics are frequently detected in different environmental media such as water bodies, soil, and sediments [13,14,15,16,17]. It was estimated that a total of 53,800 tons of antibiotics were released into the aquatic environment in 2013 [12]. Antibiotics can be released into the open environment in a variety of ways, such as wastewater treatment plant effluents, industrial effluents, and surface runoff from agriculture and animal husbandry [18,19]. Among these, the wastewater discharged from the wastewater treatment plants was a key pathway for antibiotics to enter the aquatic environment. In particular, studies indicate that the concentration of target antibiotics in effluents from pharmaceutical plants was much higher than those of household, hospital, and livestock effluents (even up to mg/L) [20]. Previously, it was reported that pharmaceutical wastewater was an important reason for the occurrence of the target antibiotics in the Xiaoqing River basin in Shandong, China, and in the water environment of Vietnam. These findings indicate the urgent need for a systematic investigation of antibiotics from pharmaceutical wastewater.

In recent decades, most studies have focused on antibiotics in wastewater treatment plants and monitoring the concentration and removal efficiency of these compounds [3,21]. The Pearl River Delta (PRD), as an important economic center region in the country, has a rapidly growing pharmaceutical industry, and, to date, some cases have been reported regarding the occurrence and removal of antibiotics from several pharmaceutical plants in China. However, the overall spatial distribution and removal performance of different treatment systems to remove antibiotics in pharmaceutical plants in the PRD are still poorly understood. Studying the occurrence of antibiotics in pharmaceutical plants in different cities in the PRD is essential to better monitor and remove antibiotics and to develop future environmental control methods.

The regional distribution and removal levels of thirty antibiotics from 15 pharmaceutical plants in different cities of the PRD were studied in the present investigation. In addition, the ecological risks of the target antibiotics in pharmaceutical plant effluents were evaluated using risk quotients (RQs). The study may provide some useful information for the design and operation of pharmaceutical plants in China for more effective antibiotic removal.

## 2. Materials and Methods

### 2.1. Regent and Materials

The thirty selected antibiotic standards were divided into four categories, including thirteen sulfonamides (SAs), five macrolides (MLs), eight quinolones (QLs), and four tetracyclines (TCs). Six isotopically labeled internal standards were used for quantitative analysis. The details are listed in Table S1.

Formic acid (99%), HPLC grade methanol, and disodium EDTA (Na_2_ETDA) were purchased from Sigma Aldrich. Their purities were analytical or above. The solid phase extraction (SPE) cartridges (HLB, 500 mg, 6CC, Waters, Milford, MA, USA) were purchased from Waters Co. Ltd. The ultra-pure water (18.2 MΩcm) was generated using the Milli-Q system in the study. The stock solutions of the target antibiotics were obtained using methanol as a solvent. The working solutions of various concentration gradients were obtained by diluting the stock solution.

### 2.2. Sample Collection

The influent and effluent samples were collected from 15 pharmaceutical plants in five representative major cities in the PRD, China, from August to September 2022. The details of each pharmaceutical plant, including the location, treatment process, type of antibiotic produced, and average daily flow are summarized in Table 1. Three samples were collected at each location. The collected samples were stored in polypropylene bottles and kept in a dark place away from light, thus avoiding the photodegradation of the antibiotics in the samples.

The samples were collected and transported back to the laboratory as soon as possible. The pretreatment work was completed within 24 h and stored at −20 °C until analysis.

### 2.3. Sample Preparation

In this study, the pretreatment of the pharmaceutical plant wastewater and sludge samples was performed based on previous studies [22]. Briefly, 1000 mL wastewater samples were extracted using Oasis HLB cartridges. The eluate was dried and then re-dissolved using 1 mL methanol. The sludge samples were extracted using ethyl acetate in a 10 mL glass centrifuge tube. The extraction procedure was repeated three times and further purified using an Oasis HLB column. The detailed wastewater and sludge sample extractions are described in the Supporting Information (Text S1).

### 2.4. Instrument Analysis

The antibiotic was analyzed using Agilent 1260 series/4000 QQQ LC-MS/MS. The column used was an Agilent ZORBAX Eclipse Plus C18 column (50 mm, 2.1 mm × 1.8 μm). The column temperature was 40 °C. The injection volume was 5 μL. Mobile phase A was methanol and Mobile phase B was pure water containing 0.1% (*v*/*v*) formic acid. The detailed mobile phase gradient procedure is shown in Table S2. 

### 2.5. Ecological Risk Assessment

The ecological risk was calculated using the risk quotient (RQ) method, where RQ is defined as the ratio of the measured environmental concentration (MEC) to the predicted no-effect concentration (PNEC). The toxicity data were obtained from the literature (Table S3). The results were divided into four groups, no risk (<0.01), low (0.01–0.1), medium (0.1–1), and high (>1.0) risks [23].

### 2.6. Quality Assurance and Quality Control (QA/QC)

The average recovery of the antibiotics in the different matrices was in the range of 72–119%. The limits of quantification (LOQ) for the antibiotics in wastewater and sludge ranged from 0.30–8.20 ng/L and 0.55–6.41 ng/g (dry weight, dw), respectively. Detailed information on the QA and QC, recovery, and LOQ for individual antibiotics is provided in the Supporting Information and Table S4.

### 2.7. Data Analysis

The sampling site maps were drawn using ArcGIS 10.6 software. The statistical analysis was performed using SPSS Statistics 26.0 software. All the figures were generated using Origin 2021 software.

## 3. Results and Discussion

### 3.1. Occurrence and Concentration of Antibiotics in Wastewater Influents

Among the 30 target antibiotics, 27 were found in the influent samples from the 15 representative pharmaceutical plants in the PRD. The frequency detection for the 27 antibiotics varied significantly across different pharmaceutical plants. Overall, 44.4% of the antibiotics were detected with a detection frequency of less than 50% in the raw influent; 22.2% of target antibiotics showed a high detection frequency of more than 75%. Among them, NOR was detected by 100%. The detection frequencies of OFL and ETM-H_2_O were above 90.0% (Table 2). In summary, the most frequently detected antibiotics included QLs, MLs, and SAs, which indicated the widespread use of the compounds in the PRD.

**Figure 1:**
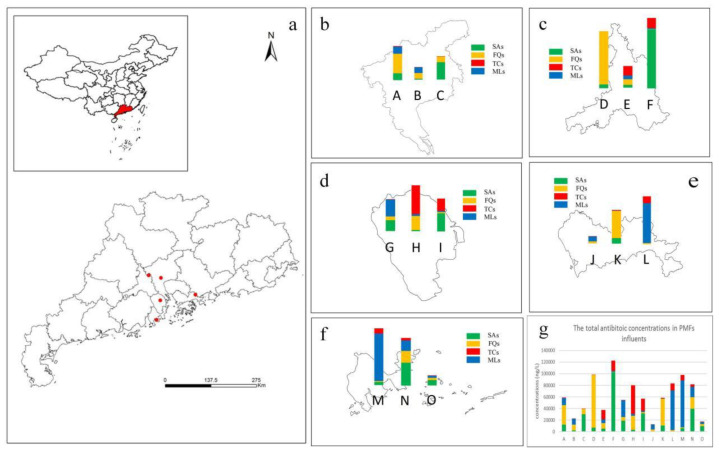
Concentration and distribution of antibiotics in influents samples from 12 pharmaceutical plants of China. (**a**): map of sampling locations. (**b**): the concentration of antibiotics in influent of Guangzhou pharmaceutical plants. (**c**): the concentration of antibiotics in influent of Foshan pharmaceutical plants. (**d**): the concentration of antibiotics in influent of Zhongshan pharmaceutical plants. (**e**): the concentration of antibiotics in influent of Shenzhen pharmaceutical plants. (**f**): the concentration of antibiotics in influent of Zhuhai pharmaceutical plants. (**g**): the total antibiotics concentration in pharmaceutical plants influent. (Abbreviations: SAs: sulfonamides; FQs: fluoroquinolones; MLs: macrolides; and TCs: tetracyclines) A–O: pharmaceutical plants A–O respectively.

The concentration of the target antibiotics ranged from a few hundred to several thousand ng/L in 15 pharmaceutical plants in the PRD (Figure 1, Table 2). The average concentration of 27 antibiotics found in the raw influents ranged from 40.33–7121.21 ng/L. In particular, SMZ, SMX, NFX, OFL CIP, LIN, ETM-H_2_O, and OTC were detected at high concentrations of 7121.21, 5338.95, 6523.78, 5140.45, 4093.42, 7671.45, 5349.97, and 4995.49 ng/L. The highest concentration of antibiotics found in the pharmaceutical plant influents was NFX, which had a median concentration of 1058.92 ng/L. In addition, the high concentrations of SMZ, SMX, OFL, CIP, LIN, OTC, and ETM-H_2_O were detected in the pharmaceutical plant influent, which had median concentrations of 102.32, 2241.33, 2489.22, 1025.31, 208.91, 2545.31, and 549.50 ng/L. It is noteworthy that the concentrations of NFX and OFL in the pharmaceutical plants of this study were usually lower than those investigated in the pharmaceutical plants in Switzerland (4562.2–10,232.3 ng/L) [24] and Croatia (5698.2–8976.2 ng/L) [25] but comparable to those in Taiwan (845.3–3465.3 ng/L) [24], China. The concentrations of SMZ were usually lower than those recorded in the pharmaceutical plants in Croatian (845.3–4320.1 ng/L) [25] and comparable to those in Guangdong (53.6–187.5 ng/L) in China [26]. The concentrations of SMX were usually higher than those in the pharmaceutical plants in Vietnam (87.3–457.4 ng/L) [27] and Tianjin (243.3–1456.2 ng/L) [28] in China but comparable to those in Europe (1737.2–2768.3 ng/L) [24]. The concentrations of SAs (SDZ, SPD, TMP, SMZ, SM, SMM, SCP, SMX, SQX, SDM, SCT, SA, and STZ) ranged from 40.33 to 7121.21 ng/L, because this kind of antibiotic was mainly used in animals. In addition to the sulfonamide antibiotics, the concentrations of the other three types of antibiotics frequently used in humans and animals were listed in the order: MLs (4073.41 ng/L) > FQs (2949.77 ng/L) > TCs (2216.32 ng/L). In addition, the concentrations of SAs, FQs, and MLs found in the investigated pharmaceutical plant influent samples were much higher than those of the target TCs in the measured wastewaters. The results suggest that SAs, FQs, and MLs are used much more frequent and in higher quantities than TCs in the PRD cities. Accordingly, the pharmaceutical plants in the PRD region mainly produce these classes of antibiotics. The result was consistent with a previous study that detected high concentrations of SA and QL antibiotics, such as SMX, NFX, and OFL, in typical antibiotics from pharmaceutical plants in the Xiaoqing River Basin, Shandong, China [22]. Moreover, according to the results of the survey, a large number of antibiotics, including QLs, SAs, MLs, and TCs, were found in the studied pharmaceutical plants. Among the target antibiotics, QLs, Sas, and MLs were predominant in the studied areas, which may be due to the high level of urbanization and dense populations with high demand for antibiotics, and the highly developed aquaculture industry in the PRD region, whose contribution to antibiotics is also not negligible.

**Figure 2:**
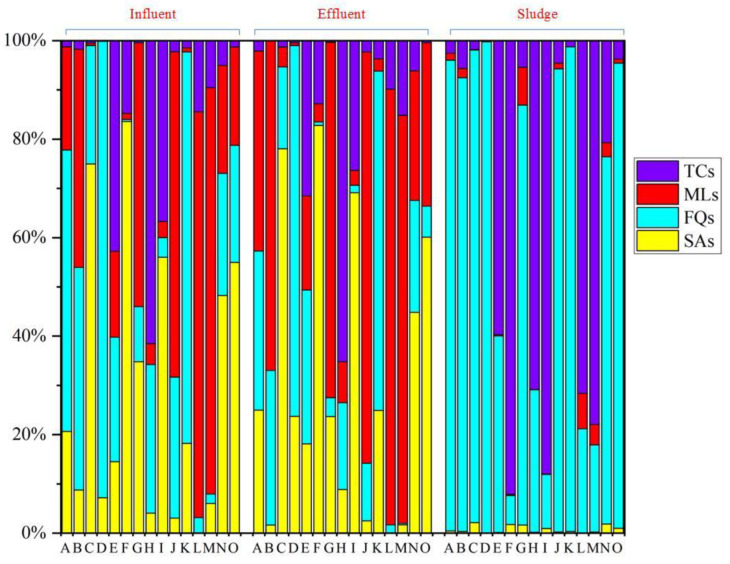
Composition profiles of different antibiotics in influent, effluent, and excess sludge from 15 pharmaceutical plants of China (Abbreviations: SAs: sulfonamides; FQs: fluoroquinolones; MLs: macrolides; and TCs: tetracyclines).

The differences in the concentration of target antibiotics in the influent water of pharmaceutical plants in different countries and regions may be due to the medication consumption per capita, water consumption per capita, GDP per capita, and antibiotic consumption preferences [29,30]. Interestingly, similar characteristics of antibiotic composition were observed in different pharmaceutical plants. As shown in Figure 2, SAs, FQs, and MLs were the major categories for antibiotics at the concentration level, accounting for from 1% (pharmaceutical plant K) to 84% (pharmaceutical plant F), from 2% (pharmaceutical plant M) to 93% (pharmaceutical plant D), and from 1% (pharmaceutical plant C) to 82% (pharmaceutical plant L), respectively, thus indicating that these types of antibiotics were found to have high production and consumption in the PRD.

**Figure 3:**
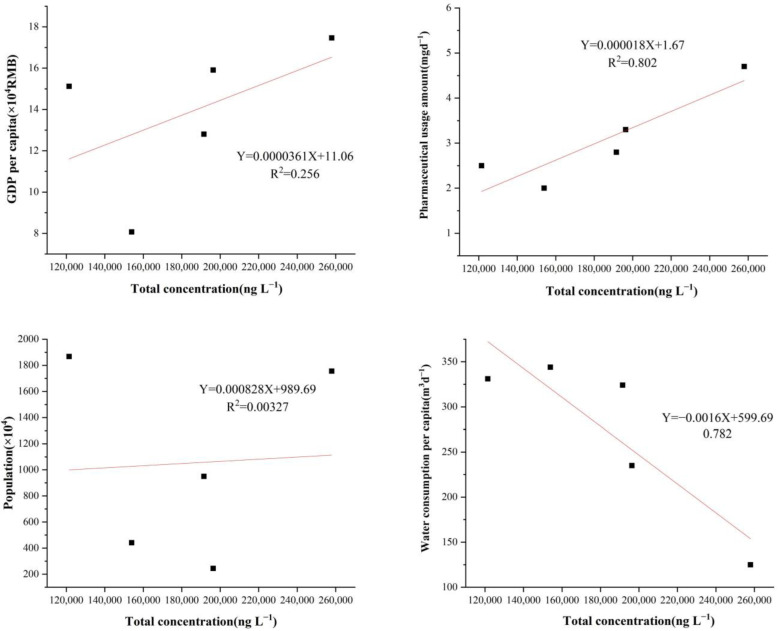
Correlation between the total concentration of 30 target antibiotics and the population served by the pharmaceutical plants, GDP per capita, water consumption per capita, and pharmaceutical usage amount per day in the regions with pharmaceutical plants.

The geographical location of the pharmaceutical plants may affect the concentration of antibiotics in the influent water to some extent (Figure 1). For example, higher total antibiotic concentrations were found in pharmaceutical plants D and F located in developed cities in the PRD (high population and density) than those of other pharmaceutical plants investigated in other studies. To better understand what factors contributed to such an uneven regional distribution of antibiotics, whether the total antibiotic concentration was associated with the population size, GDP per capita, water consumption per capita, and pharmaceutical usage amount per capita in the studied regions where the selected pharmaceutical plants were located, was analyzed in the study. The poor correlation between the total concentration of antibiotics in the influents of pharmaceutical plants and the population size of the selected pharmaceutical plants was found in the present investigation. (R^2^ = 0.00327) (Figure 3). In addition, no significant correlation between the concentration of antibiotics in the pharmaceutical plant influents and the GDP per capita of the investigated regions was found in this study. In conclusion, the concentration of antibiotics in the influents of pharmaceutical plants was not substantially affected by the population size and GDP per capita. However, high correlation coefficients with R^2^ values of 0.782 and 0.802 were found between the total influent antibiotic concentration and per capita water consumption and per capita drug use, respectively. Interestingly, there was a significant negative correlation between the total antibiotic concentrations and per capita water consumption, with higher per capita water consumption being associated with lower influent antibiotic concentrations instead. Therefore, the different total input concentrations and classes of antibiotics in the studied pharmaceutical plants exhibited a large variation, probably due to the different use and habits of antibiotics in the investigated regions.

### 3.2. Concentrations of Antibiotics in Pharmaceutical Plant Effluent and Excess Sludge

Ten antibiotics with detection frequencies over 60% were detected in effluents from pharmaceutical plants in the PRD, in which SDZ, TMP, SMZ, SMX, NFX, OFL, CIP, ETM-H_2_O, OTC, and TC showed their detection frequencies of 80%, 73%, 67%, 80%, 100%, 93%, 80%, 93%, 67%, and 67%, respectively. Moreover, the median concentrations of SDZ, TMP, SMZ, SMX, NFX, OFL, CIP, ETM-H_2_O, OTC and TC were 102.62, 56.91, 72.31, 345.92, 245.32, 556.32, 163.22, 1820.51, 278.22, and 356.22 ng/L, respectively (Table 1). Notably, the residual concentrations of SMZ in pharmaceutical plant effluents were generally lower than those recorded in pharmaceutical plants in the Xiaoqing River Basin, Shandong (127.1–7127.0 ng/L) [22], but comparable to those recorded in pharmaceutical plants of Croatian (6.7–231.0 ng/L) [25]; The residual concentrations of NFX, OFL and CIP were generally much lower than those recorded at pharmaceutical plants near Hyderabad, India (150–31,000 × 10^3^ ng/L) [31], but higher than those recorded at pharmaceutical plants in Vietnam (N.D) [27] and Taiwan (N.D) [31]. The residual concentrations of OTC and TC were higher than those recorded at pharmaceutical plants in the Xiaoqing River Basin, Shandong (N.D-12.9 ng/L), but higher than those in recorded concentrations in pharmaceutical plants in Croatia (7.4–29.3 ng/L) [25] and Taiwan (N.D-150 ng/L) [31]. The composition of antibiotics in the effluent of the pharmaceutical plant is shown in Figure 2. At the concentration level, SAs, QLs and MLs were the major antibiotic species, accounting for 83% (pharmaceutical plant F), 75% (pharmaceutical plant D) and 83% (pharmaceutical plant L), respectively. Notably, the detection frequencies of target antibiotics in the excess sludge were similar to those in the effluent samples, as shown in Table 1. Eleven of the 27 target antibiotics with detection frequencies greater than 60% were detected in the excess sludge specifically, the detection frequency of NFX was 100%, while the remaining antibiotics SDZ, TMP, SMZ, SMX, OFL, CIP, ETM-H_2_O, LIN, OTC and TC were found with detection frequencies of 80%, 73%, 67%, 80%, 93%, 80%, 93%, 67%, 67% and 67%, respectively. The highest concentrations of antibiotics in the sludge were QLs, which included OFL, NFX and CIP, with median concentrations of 4789.61, 3256.22, and 2456.11 ng/g, respectively. (Table 1 and Figure 2). TCs were also present in high concentrations in the sludge, which included OTC and TC, with median concentrations of 785.62 and 879.22 ng/g, respectively. The QLs, including OFL, and NFX, were comparable to those detected in wastewater treatment plants (WWTPs) in southern China (2014.23–6057.43 ng/g) [32], but higher than those detected in northeastern WWTPs (198.02–657.67 ng/g) [33]. The concentrations of TCs, including OTC and TC, were lower than the concentrations detected in WWTPs in South China (1756.21–3982.43 ng/g) [32]. Notably, QLs and TCs were the major antibiotics in the remaining sludge, accounting for 98% (pharmaceutical plant K) and 92% (pharmaceutical plant F), respectively (Figure 2).

### 3.3. Aqueous Removal Efficiency of Antibiotics

**Figure 4:**
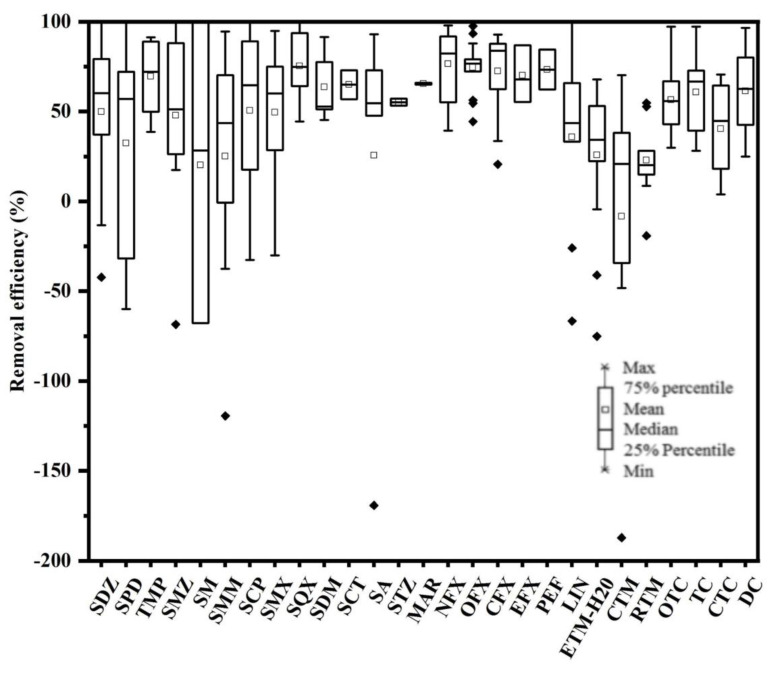
Removal efficiencies of 30 individual antibiotics in the pharmaceutical plants (Abbreviations: SDZ: Sulfadiazine; SPD: Sulfapyridine; TMP: Trimethoprim; SMZ: Sulfamethazine; SM: Sulfameter; SMM: Sulfamonomethoxine; SCP: Sulfachlorpyridazine; SMX: Sulfamethoxazole; SA: Sulfadoxine; SQX: Sulfaquinoxaline; SDM: Sulfadimethoxine; SCT: Sulfacetamide; STZ: Sulfathiazole; MAR: Marbofloxacin; NFX: Norfloxacin; OFL: Ofloxacin; CIP: Ciprofloxacin; EFX: Enrofloxacin; PEF: Pefloxacin; LIN: Lincomycin; ETM-H_2_O: Erythromycin-H_2_O; CTM: Clarithromycin; RTM: Roxithromycin; OTC: Oxytetracycline; TC: Tetracycline; CTC: Chlorotetracycline; and DC: Doxycycline).

After treatment during the pharmaceutical plant process, a large number of antibiotics remained in the pharmaceutical plant wastewater, and the removal efficiencies of the 15 pharmaceutical plants in the PRD for 27 antibiotics varied widely. The box line plots showed the removal efficiencies of the target antibiotics from 15 pharmaceutical plants in the PRD (Figure 4), which were calculated from the concentrations of the targets antibiotics in the wastewater. High removal rates for each antibiotic were found in pharmaceutical plants effluent treatment process: 76.54 ± 12.8% for NFX, 75.31 ± 10.4% for SQX, 74.54 ± 4.8% for OFL, 72.52 ± 5.8% for CIP, and 71.2 ± 9.3% for PEF. Similarly, a high removal efficiency for pharmaceutical plants was reported in previous studies [22,29]. As an example, removal rates of 93.9% and 100% for NFX and PEF, respectively, were found in a pharmaceutical plant in the Xiaoqing River Basin in Shandong, China [22]. 

**Figure 5:**
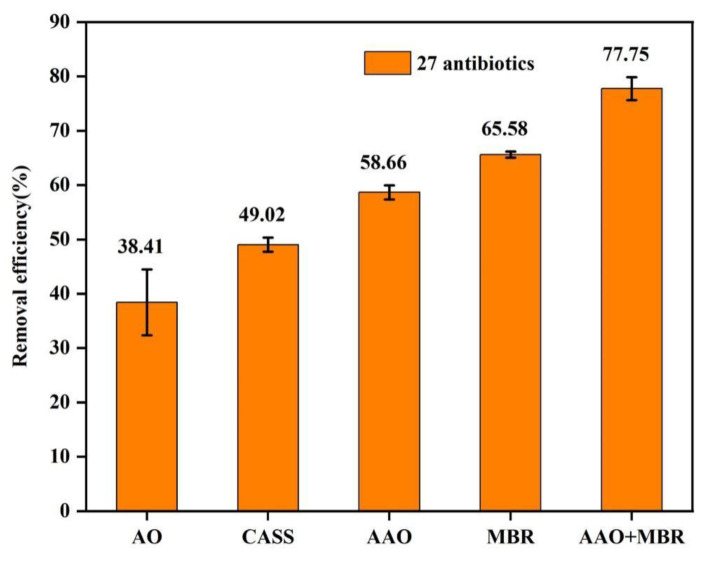
Mean removal efficiencies (%) of antibiotics in pharmaceutical plants with different treatment processes (Abbreviations: AO: anaerobic/aerobic; CASS: conventional activated sludge system; AAO: anaerobic/anoxic/aerobic; MBR: membrane bioreactor; and AAO + MBR: anaerobic/anoxic/aerobic and membrane bioreactor).

To determine whether the performance of different pharmaceutical plants for antibiotic removal was related to the adopted treatment process, 15 pharmaceutical plants were divided into five groups according to the type of wastewater treatment process adopted. The total antibiotic removal efficiency for each plant was compared in the study. The different treatment processes were adopted in different groups of pharmaceutical plants, which included recirculating activated sludge (CASS), anaerobic/aerobic (AO), anaerobic/anoxic/aerobic (AAO), sequencing batch reactor (SBR), and AAO membrane bioreactor (MBR)-integrated processes. The results showed that the average removal rate of the antibiotics varied significantly in the pharmaceutical plants with different secondary treatment processes. The biological treatment processes used in the different pharmaceutical plants are shown in Table 1. Three pharmaceutical plants had overall removal efficiencies of more than 75% for antibiotics, including pharmaceutical plant C (75.88%), pharmaceutical plant D (81.98%), and pharmaceutical plant K (75.40%). Five pharmaceutical plants had overall removal efficiencies of less than 50% for antibiotics, including pharmaceutical plant B (47.84%), pharmaceutical plant G (45.64%), pharmaceutical plant I (43.24%), pharmaceutical plant L (47.61%), and pharmaceutical plant O (26.34%), which contradicted the low removal efficiencies detected for most of the individual antibiotics (only 2 of the 27 antibiotics were removed by over 75%). Specifically, SQX and NFX exhibited high removal efficiencies of 75.31% and 76.54%, respectively. The studies reported that NFX could be almost completely removed due to its positively charged surface, which was highly susceptible to being adsorbed by negatively charged sludge, and NFX with high removal efficiencies of more than 90%, which was much higher than those in the present study [33]. In our study, 8 of the 27 antibiotics had low removal efficiencies of from −8.45% to 34.73%, while only 5 antibiotics had removal efficiencies higher than 70%. In addition to NFX and SQX, the other three effectively removed antibiotics included OFL (74.54%), CIP (72.52%), and PEF (73.37%). On the one hand, the removal rates of the total and individual antibiotics showed a large difference, mainly due to some antibiotics that had high concentrations and high removal efficiencies, thus increasing the overall antibiotic removal efficiency and leading to an overestimation of the removal effect. On the other hand, a small number of antibiotics with high concentrations and low removal efficiencies decreased the overall antibiotic removal efficiency and led to an underestimation of the removal effect. In this study, we investigated the removal efficiency of antibiotics in five groups of pharmaceutical plants with different processes, the results showed that the MBR system exhibited better removal performance than several other process systems in eliminating the antibiotics detected in the wastewater in pharmaceutical plants using a single treatment process (Figure 5). For example, compared to pharmaceutical plants using AO, CASS, and AAO systems, the MBR systems were able to remove most of the detected antibiotics, with SAs, FQs, and MLs predominating, while negative removals were often observed in CASS and AAO systems. MBR showed higher removal efficiency than other systems in treating sulfonamide antibiotics (SMZ and SQX) in pharmaceutical plants, probably due to biodegradation, which allowed the microorganisms in the MBR system to achieve sufficient degradation of antibiotics due to the longer sludge retention time [34,35]. Sludge particles may also be involved in the biodegradation process, as particles can increase the adsorption of antibiotics by the reinforced system [36]. In addition, the MBR showed strong performance in removing certain FQ class antibiotics (e.g., NFX and OFL). The efficiencies of the AAO and CASS systems for the removal of antibiotics was analyzed, and the AAO system was slightly better than the CASS system. In addition, pharmaceutical plants that adopted the AO process exhibited the worst removal rates (26.34–45.64%), which may be related to the shorter hydraulic retention time of AO [37,38]. Interestingly, it was also found that the combined process of AAO + MBR showed significantly better performance than single treatment processes such as AO, CASS, MBR, and AAO in removing most of the antibiotics from the wastewater. The results showed that the pharmaceutical plant with a combined wastewater treatment process (AAO + MBR) had significantly higher removal rates for antibiotics than the pharmaceutical plant with a single wastewater treatment process for antibiotics removal, which may be related to the biodegradation of slowly growing functional microorganisms and sludge adsorption in both systems, due to a significant increase in the sludge residence time and prolonged residence time of antibiotics in the membrane bioreactor, leading to their removal due to high adsorption and biodegradation rates. It was also found that the removal rates of different types of antibiotics by the same process varied greatly, with QLs and TCs showing high removal effects due to their easy adsorption by sludge and easy degradation by microorganisms. On the contrary, Sas and MLs showed low removal rates because they were not easily adsorbed and degraded, and some MLs even showed negative removal, which may be due to the fact that these antibiotics were ingested by humans, excreted in the form of feces, and re-released into the wastewater system through wastewater flushing and biodegradation, thus leading to an increase in the concentration of these antibiotics in the wastewater. These results suggested that the treatment process had a non-negligible influence on the antibiotic removal performance. In addition, the half life, photodegradation, and oxidative degradation properties are also important factors contributing to the removal of antibiotics in pharmaceutical plants [39,40,41].

### 3.4. Ecological Risk Assessment

**Figure 6:**
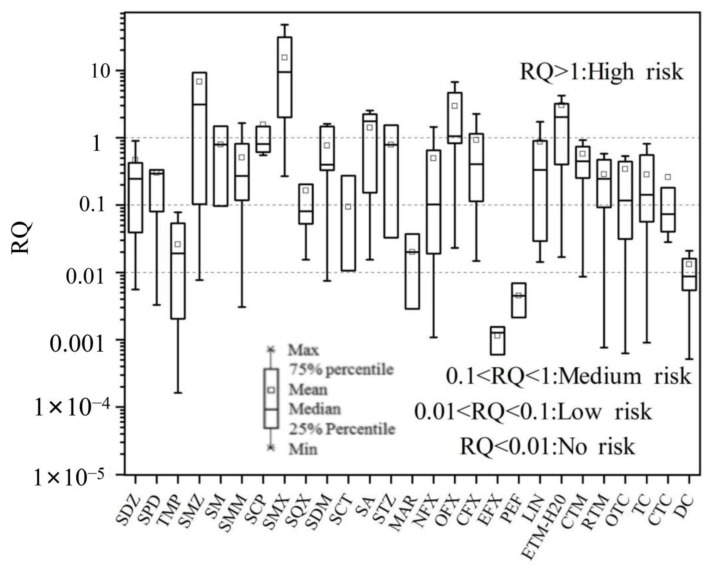
Risk quotients (RQs) for 27 target antibiotics in the pharmaceutical plant effluents (Abbreviations: SDZ: Sulfadiazine; SPD: Sulfapyridine; TMP: Trimethoprim; SMZ: Sulfamethazine; SM: Sulfameter; SMM: Sulfamonomethoxine; SCP: Sulfachlorpyridazine; SMX: Sulfamethoxazole; SA: Sulfadoxine; SQX: Sulfaquinoxaline; SDM: Sulfadimethoxine; SCT: Sulfacetamide; STZ: Sulfathiazole; MAR: Marbofloxacin; NFX: Norfloxacin; OFL: Ofloxacin; CIP: Ciprofloxacin; EFX: Enrofloxacin; PEF: Pefloxacin; LIN: Lincomycin; ETM-H_2_O: Erythromycin-H_2_O; CTM: Clarithromycin; RTM: Roxithromycin; OTC: Oxytetracycline; TC: Tetracycline; CTC: Chlorotetracycline; and DC: Doxycycline).

After treatment by the wastewater treatment system of pharmaceutical plants, the concentration of certain antibiotics in the effluent was still high and may pose a non-negligible risk to the aquatic environment. Since it was difficult to eliminate all antibiotics, priority controls must be implemented for some contaminants with high risk. To evaluate the potential ecological risk of target antibiotics in wastewater from the pharmaceutical industry in the PRD, the RQ of 27 antibiotics in pharmaceutical plant wastewater were evaluated (Figure 6). For risk of target antibiotics, SMZ, SM, SMM, SCP, SMX, SDM, SA, STZ, NFX, OFL, CIP, LIN, and ETM-H_2_O may adversely affect aquatic organisms at more than one sampling site (RQ > 1). The occurrence probabilities of high risk caused by SMX, OFL, and ETM-H_2_O in the 15 pharmaceutical plants were 73.3%, 53.3%, and 60%, respectively, which implied a high ecological risk of target pollutants. Therefore, these compounds must be marked as priority pollutants for control by the pharmaceutical plant industry in China.

Previous studies have shown that SMX, OFL, and ETM-H_2_O pose a huge environmental risk to aquatic organisms [42] Four antibiotics, including SDZ, SMZ, NFX, and CIP posed at least a medium risk in most pharmaceutical plants. The occurrence probability of posing medium or above risk by these compounds in the 15 pharmaceutical plants was 53.3%, 53.3%, 53.3%, and 60%, respectively. Some similar findings were reported by Wang and Zhang [22,43], who found that QLs such as NFX and CIP can pose a medium or high risk to aquatic organisms. In this study, only a few antibiotics showed very low or no risk, such as TMP, EFX, and PEF, which may be related to the fact that the corresponding antibiotics were not produced in large quantities by pharmaceutical plants in the studied regions. Nevertheless, the mixed toxicity of these target antibiotics and the long-term environmental effects of antibiotics were not mentioned in this paper and deserve further study.

## 4. Conclusions

The spatial distribution and removal efficiency of antibiotics in 15 pharmaceutical plants in the PRD areas were studied in this paper. The concentrations of target antibiotics varied significantly among different antibiotic species and pharmaceutical plants that adopted different biological treatment processes. The total concentration of antibiotics in the raw influents of the Shenzhen pharmaceutical plants was significantly higher than the total concentration of antibiotics in the influents of pharmaceutical plants in other investigated areas of the PRD. Among the 27 antibiotics detected in pharmaceutical plant influents, SAs, FQs, and MLs were the major contaminants, including SMX, NFX, OFL, and ETM-H_2_O. The removal efficiencies of target antibiotics varied significantly in the 15 typical pharmaceutical plants that adopted different wastewater treatment processes. In the case of a single treatment process, the MBR exhibited better removal efficiencies of antibiotics than other treatment processes. Interestingly, a higher removal efficiency of antibiotics was found in the AAO + MBR combination process than in the other single treatment processes due to the long sludge retention time of both systems, which allowed antibiotics to be removed by adequate adsorption and biodegradation. A large amount of antibiotics remained in the pharmaceutical plant effluents, among which SMX, OFL, ETM-H_2_O, SDZ, SMZ, NFX, and CIP posed high or medium ecological risk. Therefore, the target pollutants must be listed as priority pollutants in future wastewater management in PRD regions. This study provided useful results for the control of antibiotics as emerging pollutants in different regions. Seasonal variation and continuous long-term monitoring of antibiotics should be paid more attention in the future.

## Figures and Tables

**Table 1:** The specific coordinates of each sampling locations.

Sample Point	Location	Average Daily Flow (m^3^ Day^−1^)	Antibiotic Products	Treatment Process
Pharmaceutical plant A	Guangzhou	500	SAs, QLs, MLs	MBR ^a^
Pharmaceutical plant B	Guangzhou	600	QLs, MLs	CASS ^b^
Pharmaceutical plant C	Guangzhou	400	SAs, QLs	AAO + MBR ^c^
Pharmaceutical plant D	Shenzhen	800	SAs, QLs	AAO + MBR
Pharmaceutical plant E	Shenzhen	600	QLs, TCs	AAO ^d^
Pharmaceutical plant F	Shenzhen	1200	SAs, TCs	MBR
Pharmaceutical plant G	Foshan	400	SAs, MLs	AO ^e^
Pharmaceutical plant H	Foshan	600	QLs, TCs	AAO
Pharmaceutical plant I	Foshan	500	SAs, TCs	AO
Pharmaceutical plant J	Zhongshan	500	QLs, MLs	CASS
Pharmaceutical plant K	Zhongshan	600	SAs, QLs	AAO + MBR
Pharmaceutical plant L	Zhongshan	400	MLs, TCs	CASS
Pharmaceutical plant M	Zhuhai	600	SAs, MLs, TCs	MBR
Pharmaceutical plant N	Zhuhai	700	SAs, QLs, MLs, TCs	AAO
Pharmaceutical plant O	Zhuhai	600	SAs, QLs, MLs	AO

^a^: Membrane bioreactor. ^b^: Conventional activated sludge system. ^c^: Anaerobic/anoxic/aerobic and membrane bioreactor. ^d^: Anaerobic/anoxic/aerobic. ^e^: Anaerobic/aerobic. Abbreviations: SAs: sulfonamides; FQs: fluoroquinolones; MLs: macrolides; and TCs: tetracyclines.

**Table 2:** Antibiotic concentrations in the influent, effluent, and excess sludge of 15 Chinese pharmaceutical plants.

Compound	Influent (ng/L, n = 15)				Effluent (ng/L, n = 15)				Excess Sludge (ng/g, n = 15)			
	Range	Mean	Median	Freq (%)	Range	Mean	Median	Freq (%)	Range	Mean	Median	Freq (%)
SDZ ^1^	<0.35–4269.31	671.31	215.61	87	<0.44–2896.32	375.34	102.62	80	<0.74–78.21	12.65	5.62	80
SPD ^2^	<0.38–1235.91	117.46	<0.38	40	<0.64–554.82	70.15	<0.64	33	<1.24–24.61	2.83	<1.24	33
TMP ^3^	<1.58–5681.12	1095.62	204.64	73	<0.45–1245.32	305.09	56.91	73	<0.94–145.32	30.26	7.92	73
SMZ ^4^	<2.43–59,825.12	7121.21	102.32	73	<2.32–23,564.32	3185.55	72.31	67	<3.61–356.22	52.36	4.31	67
SM ^5^	<1.36–2489.52	173.24	<1.36	20	<1.58–1786.61	126.86	<1.58	13	<2.98–48.92	3.59	<2.98	20
SMM ^6^	<2.4–3895.67	619.14	83.65	53	<12.3–2489.62	406.56	4.61	53	<4.8–45.71	8.29	2.61	53
SCP ^7^	<5.6–5968.32	1075.22	<5.6	40	<5.6–3489.42	416.31	<5.6	33	<6.4–123.62	18.41	<6.4	33
SMX ^8^	<0.54–28,456.52	5338.95	2241.33	80	<0.56–7156.32	1858.44	345.92	80	<1.24–541.22	87.01	24.56	80
SQX ^9^	<0.25–1452.30	154.04	<0.25	40	<1.59–325.61	38.18	<1.59	33	<0.55–24.36	2.89	<0.55	40
SDM ^10^	<0.58–4756.21	830.15	<0.58	33	<0.84–2248.91	356.62	<0.84	33	<0.94–104.22	18.71	<0.94	33
SCT ^11^	<3.2–569.31	40.33	<3.2	13	<2.46–245.32	16.99	<2.46	13	<3.42–8.32	0.64	<3.42	13
SA ^12^	<0.96–5789.32	1125.91	<0.96	40	<8.6–2036.51	452.05	<8.6	40	<1.89–58.42	10.73	<1.89	40
STZ ^13^	<1.24–2875.69	195.46	<1.24	13	<1.21–1233.51	83.99	<1.21	13	<1.57–54.32	3.72	<1.57	13
ΣSAs	26.91–102,413.82	18,557.77	9366.81	NA	5.61–35,732.22	7692.11	4192.12	NA	2.32–936.06	252.11	171.45	NA
MAR ^14^	<2.4–2563.31	184.46	<2.4	13	<3.4–895.61	64.33	<3.4	13	<4.8–5485.22	397.57	<4.8	13
NFX ^15^	<0.61–52,302.91	6523.78	1058.92	100	<4.8–6339.81	1186.23	245.32	100	<6.4–57,824.11	8853.54	3256.22	100
OFL ^16^	<0.54–28,548.22	5140.45	2489.22	93	<0.68–7452.31	1457.44	556.32	93	<1.24–34,582.22	9096.35	4789.61	93
CIP ^17^	<3.4–28,756.52	4093.42	1025.31	80	<2.8–4782.31	810.69	163.22	80	<5.6–30,452.11	6306.53	2456.11	80
EFX ^18^	<2.9–3489.22	359.98	<2.9	20	<3.4–556.92	81.83	<3.4	20	<4.2–6893.22	812.72	<4.2	20
PEF ^19^	<0.25–18,569.23	1396.51	<0.25	13	<0.47–2890.32	252.45	<0.47	13	<0.55–21,563.42	1876.31	<0.55	13
ΣFQs	519.22–90,941.42	17,698.59	9408.22	NA	107.72	3852.97	1597.92	NA	2262.54–125,761	27,343.01	21,737.8	NA
LIN ^20^	<1.45–56,258.34	7671.45	208.91	67	<2.8–30,256.31	3752.13	104.61	60	<3.42–1025.31	158.59	14.51	67
ETM-H_2_O ^21^	<0.89–38,952.31	5349.97	2545.31	93	<0.79–12,489.12	2657.29	1820.51	93	<1.45–689.22	153.18	51.32	93
CTM ^22^	<0.69–12,895.52	1866.36	12.64	53	<0.94–7105.62	1282.31	36.22	53	<2.52–569.32	68.33	5.41	53
RTM ^23^	<0.58–4756.52	1405.83	609.62	60	<0.68–3489.32	1025.49	452.51	60	<0.94–785.33	117.28	14.31	60
ΣMLs	61.54–80,628.52	16,293.61	6489.51	NA	102.52–38,589.22	8717.22	4166.14	NA	3.64–2108.04	497.39	124.32	NA
OTC ^24^	<3.2–38,456.22	4995.49	549.50	67	<4.2–15,289.32	2021.55	278.22	67	<3.42–45,789.32	8314.19	785.62	67
TC ^25^	<0.96–15,463.52	3291.25	536.22	67	<2.14–5124.62	1185.57	356.22	67	<1.89–31,458.22	6397.82	879.22	67
CTC ^26^	<1.24–2896.51	321.09	<1.24	33	<1.08–2369.51	210.81	<1.08	33	<1.57–5489.24	727.63	<1.57	33
DC ^27^	<3.7–1356.32	257.43	104.56	53	<4.9–552.91	87.31	6.42	53	<4.2–3452.32	579.41	320.32	53
ΣTCs	104.56–49,398.11	8865.27	861.01	NA	11.34–20,859.53	3505.25	535.42	NA	320.34–68,955.92	16,019.05	1331.52	NA

^1^ Sulfadiazine; ^2^ Sulfapyridine; ^3^ Trimethoprim; ^4^ Sulfamethazine; ^5^ Sulfameter; ^6^ Sulfamonomethoxine; ^7^ Sulfachlorpyridazine; ^8^ Sulfamethoxazole; ^9^ Sulfaquinoxaline; ^10^ Sulfadimethoxine; ^11^ Sulfacetamide; ^12^ Sulfadoxine; ^13^ Sulfathiazole; ^14^ Marbofloxacin; ^15^ Norfloxacin; ^16^ Ofloxacin; ^17^ Ciprofloxacin; ^18^ Enrofloxacin; ^19^ Pefloxacin; ^20^ Lincomycin; ^21^ Erythromycin-H_2_O; ^22^ Clarithromycin; ^23^ Roxithromycin; ^24^ Oxytetracycline; ^25^ Tetracycline; ^26^ Chlorotetracycline; and ^27^ Doxycycline.

## Data Availability

Data is saved in the Supplementary Materials.

## Supplementary Materials

The following supporting information can be downloaded at: https://www.mdpi.com/article/10.3390/toxics11040382/s1, Table S1: Chemicals and internal standards; Table S2: HPLC working conditions for quantification of the target antibiotics; Table S3: PNECs of the detected antibiotics for different living organisms in waters; Table S4: Average recoveries, limit of detection (LOD), and limit of quantification (LOQ) of seventeen target antibiot-ics for wastewater samples.
