# Supplementary material for: Spatial Distribution and Risk Assessment of Antibiotics in 15 Pharmaceutical Plants in the Pearl River Delta
Source: Toxics. 2023 Apr 17;11(4):382. doi: 10.3390/toxics11040382 (PMC10143516; doi:10.3390/toxics11040382)
Supplement: Supplementary file 1 [file toxics-11-00382-s001.zip › toxics-2288991-supplementary.pdf]

Supplementary Information

Spatial distribution and risk assessment of antibiotics in 15 pharmaceutical plants in the Pearl River Delta

Yuanfei Liu[1,2], Xiaoxia Shi[1], Xiaoxia Chen[1,3], Ping Ding[1], Lijuan Zhang[1], Jian Yang[1], Jun Pan[1,3], Yunjiang Yu[1], Jinhua Wu[2], Guocheng Hu[1*]

[1]State Environmental Protection Key Laboratory of Environmental Pollution Health Risk Assessment, South China Institute of Environmental Sciences, Ministry of Ecology and Environment, Guangzhou 510535, China

[2]School of Environment and Energy, South China University of Technology, Guangzhou 510641, China

[3]Chongqing Key Laboratory of Water Environment Evolution and Pollution Control in Three Gorges Reservoir, Chongqing Three Gorges University, Chongqing 404000, China

[*]Corresponding author

E-mail: huguocheng@scies.org (GH)

Text S1. Pre-treatment methods for wastewater and sludge samples

The solid phase extraction (SPE) was used to extract target antibiotics from wastewater samples according to a previous study. First, 1000 mL of wastewater samples was filtered through a 0.47 μm glass fiber membrane. The Na_2EDTA was added to the wastewater samples to inhibit complexation of target antibiotics with other metal ions, thus improving the recovery of the substances to be measured. All wastewater samples were acidified with 4 M sulfuric acid to pH=3.0. Before pretreatment, internal standard (100 ng/L) was added to each sample for the quantitative analysis of each analyte. Oasis HLB cartridges were activated sequentially with 10 mL methanol and 10 mL ultrapure water in turn. Subsequently, all water samples were passed through solid phase extraction column at a low flow rate. After extraction, the cartridges were then rinsed with 3×5 mL ultrapure water and then drained under vacuum for an hour. Next, the target antibiotics in the cartridge were eluted with 5 mL methanol, 4 mL ethyl acetate, and 3 mL dichloromethane. The analytes were then concentrated under a stream of nitrogen until the methanol was almost completely evaporated. Finally, the sample is redissolved in 1 mL methanol and stored in a brown sample bottle for analysis.

Table S1 Chemicals and internal standards

Antibiotics	Abbr.[a]	Group	ISTD[b]
Sulfadiazine	SDZ	sulfonamides (SAs)	sulfamethoxazole-D_4
Sulfapyridine	SPD	sulfonamides (SAs)	sulfamethoxazole-D_4
Trimethoprim	TMP	sulfonamides (SAs)	sulfamethoxazole-D_4
Sulfamethazine	SMZ	sulfonamides (SAs)	sulfamethazine-$^{13}C_6$
Sulfameter	SM	sulfonamides (SAs)	sulfamethazine-$^{13}C_6$
Sulfamonomethoxine	SMM	sulfonamides (SAs)	sulfamethoxazole-D_4
Sulfachlorpyridazine	SCP	sulfonamides (SAs)	sulfamethoxazole-D_4
Sulfamethoxazole	SMX	sulfonamides (SAs)	sulfamethoxazole-D_4
Sulfadoxine	SA	sulfonamides (SAs)	sulfamethoxazole-D_4
Sulfaquinoxaline	SQX	sulfonamides (SAs)	sulfamethoxazole-D_4
Sulfadimethoxine	SDM	sulfonamides (SAs)	sulfamethoxazole-D_4
Sulfacetamide	SCT	sulfonamides (SAs)	sulfamethoxazole-D_4
Sulfathiazole	STZ	sulfonamides (SAs)	sulfamethazine-$^{13}C_6$
Marbofloxacin	MAR	fluoroquinolones (FQs)	ciprofloxacin-D_8
Norfloxacin	NFX	fluoroquinolones (FQs)	ciprofloxacin-D_8
Ofloxacin	OFL	fluoroquinolones (FQs)	ciprofloxacin-D_8
Ciprofloxacin	CIP	fluoroquinolones (FQs)	ciprofloxacin-D_8
Enrofloxacin	EFX	fluoroquinolones (FQs)	ciprofloxacin-D_8
Pefloxacin	PEF	fluoroquinolones (FQs)	ciprofloxacin-D_8
Lincomycin	LIN	macrolides (MLs)	erythromycin-^{13}C-D_3
Erythromycin-H_2O	ETM-H_2O	macrolides (MLs)	erythromycin-^{13}C-D_3
Clarithromycin	CTM	macrolides (MLs)	erythromycin-^{13}C-D_3
Roxithromycin	RTM	macrolides (MLs)	erythromycin-^{13}C-D_3
Oxytetracycline	OTC	tetracyclines (TCs)	meclocycline
Tetracycline	TC	tetracyclines (TCs)	meclocycline
Chlorotetracycline	CTC	tetracyclines (TCs)	meclocycline
Doxycycline	DC	tetracyclines (TCs)	meclocycline

a. Abbr.: abbreviation;

b. ISTD: internal standard chemicals;

Table S2. HPLC working conditions for quantification of the target antibiotics

Parameter	SAs, FQs, MLs, TCs ESI+
Column temperature (°C)	40
Flow rate (mL/min)	0.30
Mobile phase A	0.2%Formic acid and 2 Mm ammonium acetate
Mobile phase B	Acetonitrile
	0 min: 10% B
	0~5 min: 15% B
	5~7 min: 20% B
Elution gradient	7~11 min: 40% B
	11~15 min: 60% B
	15~16 min: 95% B
	16~25 min: 95% B

Table S3. PNECs of the detected antibiotics for different living organisms in waters

Compounds	PNECs (ng/L)		
	Freshwater algae	Invertebrates *(Daphnia)*	Fish (zebrafish embryos)
SDZ	40390	1000	238300
SPD	20790	700	45400
TMP	N.D	16000	N.D
SMZ	19470	700	32600
SM	1200	N.D	N.D
SMM	4750	1500	N.D
SCP	28080	800	78900
SMX	150	252000	562500
SQX	700	15000	N.D
SDM	9850	2259	166297
SCT	8562	900	71903
STZ	1600	800	10000
MAR	30000	24000	60000
NFX	4750	2400	9830
OFL	1000	530	6500
CIP	2500	1100	8700
EFX	N.D	357900	N.D
PEF	N.D	419700	N.D
LIN	70	7200	1040222
ETM-H_2O	2300	940	10000000
CTM	1400	4200	4990
LIN	70	7200	1040222
RTM	4660	6000	23000
OTC	12500	8900	65100
TC	8800	6270	N.D
CTC	5000	2450	N.D
DC	25000	12400	95000

The acute and chronic toxicity data were derived from literatures [3-7].

PNECs was calculated from the toxicity data using an assessment factor (AF) of 1000 for acute toxicity and an AF of 100 for chronic toxicity.

N.D., no data reported.

Table S4. Average recoveries, limit of detection (LOD) and limit of quantification (LOQ) of seventeen target antibiotics for wastewater samples

Antibiotics	Influent			Secondary effluent			Sludge		
	Average recoveries (%)	LOD (ng/L)	LOQ (ng/L)	Average recoveries (%)	LOD (ng/L)	LOQ (ng/L)	Average recoveries (%)	LOD (ng/g)	LOQ (ng/g)
SDZ	69.1±5.8	0.39	1.29	79.1±4.2	0.44	1.29	74.2±3.8	0.74	2.35
SPD	95.9±9.8	0.31	1.03	94.7±5.6	0.64	1.92	98.0±5.1	1.24	3.72
TMP	96.0±6.2	0.29	0.96	96.2±5.4	0.45	1.35	123.4±13.6	0.94	3.12
SMZ	100.7±11.6	0.57	1.87	105.7±12.6	2.32	6.98	98.8±7.9	3.61	10.84
SM	74.2±3.8	0.30	0.98	78.4±4.5	1.58	4.77	91.4±13.7	2.98	9.32
SMM	98.0±5.1	10.71	33.2	99.2±5.3	12.3	36.8	108.2±11.9	4.8	15.2
SCP	123.4±13.6	5.21	16.2	125.7±11.5	5.6	16.8	99.6±7.2	6.4	19.5
SMX	98.8±7.9	0.25	0.82	86.5±7.8	0.56	1.68	106.6±9.7	1.24	3.72
SA	88.8±6.9	0.45	1.35	84.5±7.8	0.46	1.54	116.6±9.7	2.24	6.72
SQX	91.4±13.7	0.52	1.73	90.2±11.7	0.59	1.84	108.1±17.3	0.55	1.65
SDM	108.2±11.9	2.2	1.02	101.2±10.4	1.84	5.78	87.3±9.9	0.94	2.98
SCT	99.6±7.2	1.02	3.37	97.6±5.4	2.46	7.56	87.3±9.9	3.42	10.21
STZ	106.6±9.7	0.21	0.7	101.2±6.5	1.21	3.68	133.1±5.8	1.89	5.74
MAR	108.1±17.3	1.1	3.63	104.5±12.4	3.4	10.2	74.2±3.8	1.57	4.85
NFX	87.3±9.9	0.89	2.93	82.5±8.2	0.8	2.45	98.0±5.1	3.4	10.23
OFL	133.1±5.8	0.29	0.96	110.1±4.5	0.68	1.95	119.4±13.6	6.4	19.4
CIP	74.2±3.8	0.30	0.98	78.2±4.2	0.8	2.45	97.8±7.9	1.24	3.69
EFX	98.0±5.1	0.71	2.33	99.5±6.5	0.4	1.32	133.1±5.8	5.6	16.8
PEF	119.4±13.6	0.21	0.7	117.1±11.7	0.47	1.47	74.2±3.8	4.2	12.6
LIN	97.8±7.9	0.25	0.82	94.1±7.5	0.48	1.48	98.0±5.1	0.55	16.5
ETM-H_2O	91.4±13.7	0.52	1.73	91.4±13.7	0.79	2.48	97.6±5.4	3.42	10.3
CTM	108.2±11.9	2.2	1.02	108.2±11.9	0.94	3.1	101.2±6.5	1.45	4.45
RTM	106.6±9.7	0.21	0.7	106.6±9.7	0.68	2.24	104.5±12.4	2.52	7.5
OTC	108.1±17.3	1.1	3.63	108.1±17.3	1.2	3.62	82.5±8.2	0.94	2.97
TC	101.1±11.3	0.23	0.39	101.1±11.3	1.14	3.45	110.1±4.5	3.42	10.41
CTC	98.2±10.3	1.0	3.3	98.2±10.3	1.08	3.54	78.2±4.2	1.89	5.8
DC	88.1±9.3	0.45	1.35	88.1±9.3	0.9	3.1	97.6±5.4	1.57	4.6
